# History of the Discovery of Modern Anæsthesia

**Published:** 1895-01

**Authors:** Thomas Fillebrown

**Affiliations:** Boston


### International Dental Journal.

Vol. XVI. January, 1895. No. 1.

### Original Communications.[1]

### HISTORY OF THE DISCOVERY OF MODERN ANÆSTHESIA.[2]

BY DR. THOMAS FILLEBROWN, BOSTON.

To-day we'll
" Poise the cause of Justice in equal scales,
Whose beam stands sure, whose rightful cause prevails."
And
" If circumstances lead me, I will find
Where Truth is hid, though it were hid indeed
Within the centre."

Shakespeare.

Fifty years ago to-day there was enacted in the city of Hartford, Connecticut, the first scene in the development of the grandest and most beneficent discovery the world has ever beheld,—the discovery of modern anæsthesia; and this during a half-century which has exceeded all other periods in the magnitude and importance of the discoveries made in science, mechanics, and medicine. We wish to-day to pay our tribute to the memory of the discoverer of this great

[1] The editor and publishers are not responsible for the views of authors of papers published in this department, nor for any claim to novelty, or otherwise, that may be made by them. No papers will be received for this department that have appeared in any other journal published in the country.

[2] Address delivered at the Memorial Celebration of the Fiftieth Anniversary of the Discovery of Anæsthesia, Philadelphia, December 11, 1894.

fact. To this end let us examine the testimony, restate the facts, and again judge their relative value.

"We can do nothing against the truth." History makes itself; to record it impartially is a difficult task.

Great discoveries and great events do not burst forth with promethean suddenness or completeness, but are the result of long periods of incubation and growth, and oftentimes await many long years of expectancy, hope, and even despair. Such was the case with the great fact of practical anæsthesia. The ancient nations waited for it, hunted for it, but died without the sight. Modern nations continued the longing search, but they caught only a glimpse of the coming day to reward them, until the middle of the present century, when Horace Wells discovered, demonstrated, and proclaimed the great blessing "which stopped pain, robbed the knife of its terrors," and made glad the heart of every sympathizer with suffering.

Insensibility to surgical operations was occasionally induced many centuries ago. Homer mentions the anæsthetic effect of nepenthe, and refers to the inhalation of a vapor of hemp. Dioscorides and Pliny record the use of mandragora. Apuleius, A.D. 125, said, "If a man has to have a limb mutilated, sawed, or burnt, he may take an ounce of mandragora wine, and while he sleeps the member may be cut off without pain or sense." In the third century, Hoa-tho, a Chinese, gave his patients a preparation ot hemp, which rendered them insensible to pain. Theodoric, in the thirteenth century, gave directions for preparing the *Spongia somnifera* for inhalations before operations. Ether was known as early as the thirteenth century, and described by Cordus in the sixteenth century, and the name ether was given it by Frobenius in 1730. In 1828, Gerardin read a paper before the Academy of Medicine of Paris describing surgical anæsthesia produced by inhaling gases. In 1800, Sir Humphry Davy made his remarkable statement, that "As nitrous oxide gas appears capable of destroying physical pain, it may probably be used to advantage during surgical operations in which no great effusion of blood takes place." The pity is that this suggestion should have lain buried under the forgetfulness of forty-five years and bear no fruit.

None of the agents used by the ancients proved practical or safe. Consequently, at the beginning of the present century practical anæsthesia remained undiscovered.

Two agents are inseparably connected with the discovery of modern anæsthesia,—protoxide of nitrogen and sulphuric ether.

Protoxide of nitrogen was discovered by Priestley, demonstrated by Davy, and practically applied by Horace Wells in 1844. Sulphuric ether was discovered in the thirteenth century, described in the sixteenth, named in the eighteenth, and practically applied to produce surgical anæsthesia in the nineteenth century. Its use was made known to the world by Morton at the Massachusetts General Hospital in 1846.

Ever since 1818 the physiological action of these drugs has been well understood. In 1849 it was well known that both would produce intoxication, and that both would lessen the severity of pain, having been repeatedly inhaled for amusement and for relief of suffering. So it was but a step from this to the attainment of surgical success, but a step no one had dared take until Wells ventured and bridged the chasm. The history of this step is simple but dramatic. For four years, though probably ignorant of Davy's suggestion, Wells had believed it possible, by the inhalation of certain gases, especially laughing-gas, to produce a degree of intoxication that would obtund the pain of surgical operations. It was also known that Wells possessed the current knowledge concerning the properties and physiological effects of both gas and ether.

At this time G. Q. Colton was delivering throughout the country popular lectures on chemistry, administering at each lecture the laughing-gas for the amusement of the audience. December 10, 1844, he lectured in Hartford, Connecticut. In the audience was Horace Wells, with his mind still occupied with the possibilities of the gas. He inhaled the gas himself; he watched its effects on others. He said to a Mr. Clark, "I believe a man may, by taking that gas, have a tooth extracted or a limb amputated and not feel any pain." Mr. S. A. Cooley inhaled the gas, and while under its influence ran against and overthrew some benches in the hall, thereby producing several severe abrasions upon his knees. When he recovered consciousness he found the skin on his limbs badly bruised and broken, and yet he had not suffered at all, and did not know of the injury he had inflicted upon himself until the spectators had called his attention to it.

Dr. Wells observed this effect of the gas, and at once determined to try it on his own person. A troublesome wisdom-tooth offered the necessary object for the experiment. At Dr. Wells's request, Colton, on the morning of the 11th of December, took a bag of the laughing-gas to Dr. Wells's office in order that he might try the experiment of rendering the extraction of his tooth painless. A number of those present the previous evening, including Colton, Wells, and Cooley, repaired to Dr. Wells's office. What occurred there is thus described by Dr. Riggs, whose office adjoined that of Dr. Wells, and who was called in to extract the tooth: "Dr. Wells, a few minutes after I went in, and after conversation, took a seat in the operating-chair. I examined the tooth to be extracted with a glass, as I generally do. Wells took the bag of gas from Mr. Colton and sat with it in his lap, and I stood by his side; Wells then breathed the gas until he was much affected by it; his head dropped back; I put my hand to his chin, he opened his mouth, and I extracted the tooth; his mouth remained open some time. I held up the tooth in the instrument that the others might see it, they standing partially back of the screen and looking on. Dr. Wells soon recovered from the influence of the gas so as to know what he was about, discharged the blood from his mouth, swung his hand, and said, 'A new era in tooth-pulling; it did not hurt me at all!' We were all much elated, and conversed about it for an hour after. We were so elated by the success of this experiment that we immediately turned our attention to the extraction of teeth by means of this agent, and continued to devote ourselves to this subject for several weeks almost exclusively."

Dr. Wells continued to use the gas freely in the practice of dentistry during the remainder of that year and the year following, and at all times when he was in the practice of his profession. Then was the deduction and suggestion made by Humphry Davy in 1800 verified by Horace Wells and the prophecy fulfilled, and practical anæsthesia became a discovered and demonstrated reality. "The fierce extremity of suffering was steeped in the waters of forgetfulness, and the deepest furrow in the knotted brow of agony was smoothed forever."[1] This event was the source of the world's knowledge of anæsthesia. All previous efforts had come to naught, but the echoes of Wells's success were soon heard around the world.

In the beginning of the present century science could not interpret the signs, and Davy's conception and prophecy of the possibilities of nitrous oxide fell upon deaf ears, and all the knowledge concerning the power of ether to produce insensibility to pain appealed to sterile minds. And even in 1844 the professional ear was not quite attuned to the sound, and could not recognize in the extraction of Dr. Wells's tooth, on the 11th of December, 1844, the key to the solution of the whole problem of anæsthesia. Even

---

[1] O. W. Holmes.

those most interested, most acute, and most observant could not in Wells's nearly successful operation before the class of the Harvard Medical School in January, 1845, apprehend the great fact that surgical anæsthesia was a possibility; nor indeed could they perceive it until it was forced upon their attention by the courage of a Morton. If, when Wells extracted the student's tooth, the surgeons of the Massachusetts General Hospital had possessed a little more of that keen perceptive power "of feeling than of seeing, of the heart than of the ear," they would have apprehended the possibilities; and the discovery of surgical anæsthesia would have been then acknowledged and the processes perfected, and no disputing claims would have arisen.

When we realize how hard it is to compel attention to a new idea, how slow is the accumulation of new facts, how gradual the growth of perception, and how great the magnitude of this subject, we cease to wonder at the slowness with which the significance of this event was appreciated.

In future years, whenever and wherever the discovery of anæsthesia is intelligently discussed with knowledge of the subject, the name of Horace Wells will be spoken with honor and gratitude, and with his name will be associated the names of John M. Riggs, G. Q. Colton, E. E. Marcy, W. T. G. Morton, James Y. Simpson, Charles F. Jackson, Oliver Wendell Holmes, and Henry J. Bigelow.

Of these, Dr. Marcy and G. Q. Colton still survive to bear witness to their part in the drama and enjoy their honors. G. Q. Colton administered the nitrous oxide gas to Dr. Wells for the first operation under anæsthesia, and reintroduced its use in 1863. Dr. Riggs performed the first operation, extracting Wells's molar tooth. Dr. Marcy suggested to Dr. Wells the use of ether instead of gas, and verified its action. Dr. Morton made the first public application of ether for surgical anæsthesia. Dr. Jackson claimed to have suggested all that Morton knew about the effects of ether, and the use of it for anæsthetic purposes. Dr. Simpson made himself and British surgery famous by the discovery of the anæsthetic power of chloroform.

Dr. Holmes suggested for this condition of insensibility the name which has become universal. He wrote to Dr. Morton as follows:

"Everybody wants to have a hand in a great invention. All I will do is to give you a hint or two as to names, or the name, to be applied to the state produced, and to the agent.

"The state should, I think, be called anæsthesia. This signifies insensibility, more particularly (as used by Linnæus and Cullen) to objects of touch. The adjective will be anæsthetic. Thus, we might say the 'state of anæsthesia,' or the 'anæsthetic state;' the means employed would be properly called the 'anti-æsthetic agent.' Perhaps it might be allowable to say 'anæsthetic agent;' but this admits of question.

"The words anti-neuric, aneuric, neuro-leptic, neuro-lepsia, seem too anatomical; whereas the change is a physiological one. I throw these out for consideration.

"I would have a name pretty soon, and consult some accomplished scholar, such as President Everett or Dr. Bigelow, Sr., before fixing upon the terms which will be repeated by the tongues of every civilized race of mankind. You could mention these words which I suggest for their consideration; but there may be others more appropriate and agreeable. Yours respectfully,

"O. W. Holmes."

Dr. Crawford W. Long, of Georgia, used ether for anæsthetic purposes three times during 1842–43, and now appears as a claimant to the discovery. But Dr. Long's connection with the subject was not mentioned until many years after the fact. He did not write a word in regard to his discovery, nor did any notice of it appear in print until 1849,—five years after Wells's discovery, and seven years after he himself had administered the ether.

How could any one, after knowing of such a boon to suffering humanity, resist for even a day the impulse to fly, " . . . on joyful wings cleaving the sky," to proclaim the coming of this great consolation to the afflicted.

Dr. R. M. Hodges wrote of this claim, "Not a physician or surgeon ever used ether because Long had used it; nor did mankind learn from him that anæsthetic inhalation for surgical purposes was possible." His claim was made after the fact, and resting on no better foundation than those claims similarly made by other aspirants for distinction: a class so numerous as to have been named by the London *Lancet* "the class of Jump-up-behinders."

Dr. H. J. Bigelow's connection with the ether discovery was important, perhaps vital to the success of anæsthesia at that time, as in more than one instance he prevented fatal results from over-anæsthesia. Although but twenty-eight years of age, and junior surgeon at the hospital, less than one year in office, he was the one whose penetration, executive ability, sagacious and active qualities of mind and body made him realize that the event of a lifetime was taking place. He made a clinical study of the subject; he made most unremitting exertion to prove the safety of ether; he practically supervised etherization during the first year of its use; he announced to the world the discovery of ether in a paper read at the Academy of Medicine, November 3, 1846. He verified the anæsthetic power of nitrous oxide in 1848. These facts have identified Dr. Bigelow with the whole subject of anæsthesia; and had he been present at the occasion of Wells's first experiment, it is not unlikely the course of events would have been materially different.

Dr. Edward Warren wrote, "To him next to the discoverer himself are the public and the world indebted for the blessings of so early receiving this great discovery."

The many volumes printed during the exciting years from 1846 to 1863 furnish a great number of statements, opinions, and facts pertaining to this discovery, most of which have ceased to be of any value.

Napoleon is said to have often let his letters lie unopened for several days, giving as a reason that in that time events would answer a greater part of them. So we find that the larger part of the statements, opinions, and arguments in regard to this subject have been answered by subsequent events. Let us contrast a few of the statements made at that time with the facts as now known.

Then some denied that nitrous oxide was an anæsthetic; to-day it is known as one of the most efficient.

In 1844, Wells claimed that the gas was safer than ether; others denied its safety; to-day it stands proven the safest and most pleasant anæsthetic agent known to the world. It was then claimed to be impracticable to use it; now its successful administration annually to more patients than all other agents combined prove its practicability. It was then claimed to be inefficient for prolonged operations; to-day it is proved equal to continuing the anæsthetic state indefinitely. Then it was contended by skilful surgeons and eminent divines "that pain was a natural protection, a necessary stimulant to the reparative process, and a Providential dispensation, and that the prevention of it was defying the Almighty;" now it amounts to inhumanity and malpractice to presume to do any severe operation without it.

Professor Charles D. Meigs, of the Jefferson Medical College of Philadelphia, as late as 1856 wrote of the "doubtful nature of any processes that the physician sets up to contravene the operation of those natural and physiological forces that the Divinity has ordained us to enjoy or suffer," and a clergyman wrote to a medical friend as follows: Anæsthesia is "a decoy of Satan, apparently offering itself to bless women; but in the end it will harden society, and rob God of the deep, earnest cries which arise in time of trouble for help."

The following facts seem to be established by indisputable, sworn testimony, and I believe are admitted by all the friends of truth.

In 1840, Dr. Wells expressed his faith in the anæsthetic power of nitrous oxide. December 11, 1844, Wells inhaled nitrous oxide and had a tooth extracted painlessly, which event immediately became known throughout the city of Hartford and vicinity. Forthwith Wells made a pilgrimage to Boston to proclaim and demonstrate his discovery; he called on Morton, and made known to him this event. Through Morton's intercession an invitation was given Dr. Wells by Dr. J. C. Warren to speak to the class of the Harvard Medical School and describe his discovery. A little later he gave the nitrous oxide to a patient and extracted a tooth for him before the same class with incomplete success. The patient cried out as with pain, but when again conscious declared he had not been hurt.

A month later Dr. Marcy suggested to Wells the use of sulphuric ether for this same purpose, and verified its effects by anæsthetizing a patient with this agent, and removing a good-sized tumor from his head without causing pain. They both discarded ether, as its odor was unpleasant and because they considered it less safe than laughing-gas.

Early in 1845, Dr. Wells administered sulphuric ether to a patient (Gaylord Wells) and extracted a tooth painlessly. In 1841, Dr. Morton was a pupil of Dr. Wells, and in 1842 they were in business together for a time in Boston. In July, 1845, Morton called on Wells in Hartford and talked with him and Dr. Riggs about the gas, and asked them for a supply. Dr. Wells referred him to Dr. Jackson for information as to the manufacture of it. Drs. Wells, Riggs, and others continued the use of gas until November 6, 1846, when chloroform and ether were substituted, and gas remained unused until 1863.

The use of nitrous oxide for anæsthetic purposes was recorded in the *Boston Medical and Surgical Journal* of June 18, 1845, as follows: "The nitrous oxide gas has been used in quite a number of cases by our [Hartford] dentists during the extraction of teeth, and has been proved by its excitement perfectly to destroy pain."

Dr. William T. G. Morton performed his first successful operation with ether September 30, 1846, and on October 16 following he administered ether to a patient at the Massachusetts General Hospital, and on the 17th to the second case. About October 20, 1846, Dr. Jackson claimed compensation from Morton for professional advice, and charged five hundred dollars. October 27, 1846, Drs. Morton and Jackson made oath to a joint discovery of a

*compound* for the prevention of pain during surgical operations, and applied for a patent. The patent was granted, and in 1863 was declared void, as such a discovery was not patentable. November 9, 1846, Dr. Morton declared it was simply sulphuric ether, not a compound as claimed in his application for a patent. In the autumn of 1847, Drs. Jackson and Morton each claimed to be the sole independent discoverer of anæsthesia, and in no wise indebted to the other, and so contended to the end. In 1847 the Paris Academy of Medicine, upon *ex parte* evidence, declared Morton and Jackson the discoverers of anæsthesia.

In January, 1848, the Parisian Medical Society, after a full hearing of evidence from both parties, voted that "To Horace Wells, of Hartford, Connecticut, United States of America, is due all the honors of having discovered and successfully applied the uses of vapors or gases whereby surgical operations could be performed without pain," and elected him an honorary member of the society.

In 1846 the surgeon of the Massachusetts General Hospital gave Morton the credit of being the discoverer of modern anæsthesia. In 1853, five years after Wells's death, Dr. C. H. Haywood, one of the surgeons present at the first operation, gave the following unqualified credit to Wells for his share in it. He closes a letter to Hon. Truman Smith with these words: "It was no Minerva born with one blow. Moreover, in analyzing the nature of the discovery, we can detect several elements which were successfully brought to light. Thus, we observe in the first period an indefinate search after some method of producing insensibility to pain. Then came a second period where great advance was made, beyond all dispute due to Horace Wells.

"This was the first important step in the history of anæsthesia. The question of priority may be easily settled. It is satisfactorily proved that Dr. Wells's experiments had established the above-mentioned points as early as 1844, though they had not determined ether the best agent, or perfected the method of administration in detail. In the third period the anæsthetic properties of certain substances were discovered. First nitrous oxide gas was tried, then sulphuric ether, then chloroform, then chloric ether. These discoveries were all made by different individuals. Now, for which of these agents and to which discoverer shall remuneration be granted? To each and for all I say, to Morton for sulphuric ether, to Dr. Simpson for chloroform, to Dr. J. C. Warren for chloric ether; but before all, let full and ample justice be done to that noble genius which first conceived the grand idea which has been the basis of all the experiments and father of all the discoveries. To the spirit of Dr. Horace Wells belongs the honor of having given to suffering humanity the greatest boon it ever received from science."

"Thus do facts maintain the majesty of truth." Argument would only weaken the evident conclusion.

Considering Wells was timid, retiring, and only twenty-seven years old when these events occurred, who can wonder that he should return from Boston disheartened; and later, when still suffering from disappointment and the ill effects of his own sacrificing experiments, meet a sad and tragic end. It is remarkable, too, that his rival claimants should both meet an almost equally tragic fate, Morton dying of apoplexy while out riding, and Jackson spending the last seven years of his life in an asylum for the insane. Thus did "the shears of Fate cut the tent-ropes of their lives."

Upon the memory of Horace Wells there remains no blot or stain; against him no charge of selfishness, dishonesty, deceit, or unfairness was ever made. He lived and died honored and respected by the people among whom he dwelt. We best quote the words of one who knew him intimately:

"He had a mind of uncommon restlessness, activity, and intelligence. He early manifested great inventive genius and mechanical talent. He was of medium height, with a head of remarkable size, complexion light, compactly built, of pleasing countenance and address, and of fine personal appearance. As a citizen he was a man of great purity of character and of generous impulses, honoring religion by his walk and conversation; as a son, he was kind and dutiful; and in his family relations an example of kindness and affection. In all these respects he was without spot."

Such a character as this sought only the legitimate emoluments of his calling and was always ready to benefit his profession and mankind. No sacrifice was too great for him to make. Very appreciative of words of encouragement, he was also very sensitive to criticism; hence it is not strange that such a spirit, so young, should quail before the derision of professors and jeers of students. Honest himself, he could not think others dishonest; just, he could not brook injustice. Being denied what he knew were his just claims, his soul was cut to the quick, and a dark veil was drawn over what promised a brilliant and useful life. We lay our wreath upon his tomb; would that we to-day might with it crown his head. Coming generations will recognize in him the martyr and the world's benefactor, and on every monument which in the future may be raised to commemorate this great event will be inscribed, —To the Discoverer of Anæsthesia,—Horace Wells.

